# Cold acclimation conditions constrain plastic responses for resistance to cold and starvation in *Drosophila immigrans*

**DOI:** 10.1242/bio.034447

**Published:** 2018-06-15

**Authors:** Ankita Pathak, Ashok Munjal, Ravi Parkash

**Affiliations:** 1 Department of Biochemistry and Genetics, Barkatullah University, Bhopal, 462026, India; 2 Department of Genetics, Maharshi Dayanand University, Rohtak, 124001, India

**Keywords:** Cold or starvation induced plastic changes, Energy metabolites, Fecundity, *Drosophila immigrans*

## Abstract

In montane *Drosophila* species, cold-induced plastic changes in energy metabolites are likely developed to cope with cold and starvation stress. Adult *Drosophila immigrans* reared at 15°C were acclimated at 0°C or 7°C for durations of up to 6 days (fed or unfed conditions). Such flies were tested for plastic changes in resistance to cold or starvation stress as well as for possible accumulation and utilization of four energy metabolites (body lipids, proline, trehalose and glycogen). Adults acclimated at 7°C revealed a greater increase in cold tolerance than flies acclimated at 0°C. Different durations of cold acclimation at 7°C led to increased level of body lipids only in fed flies which were utilized under starvation stress. However, such plastic responses were not observed in the flies acclimated at 0°C, which remained unfed due to chill-coma. These observations suggest a possible role of feeding to improve starvation resistance only in the flies acclimated at 7°C with food. Cold acclimated *D. immigrans* flies revealed improved cold resistance through a possible reshuffling of trehalose and glycogen; and starvation-induced proline which was utilized under cold stress durations. Finally, greater reduction in mean daily fecundity due to cold or starvation was observed in 0°C acclimated flies as compared to 7°C acclimated flies. Thus, cold acclimation conditions (0°C or 7°C) greatly impact resistance to cold and starvation in *D. immigrans*.

## Abstract (precis)

**Summary:** *Drosophila immigrans* flies acclimated at 7°C (on food) showed an increase in body lipids and starvation resistance but there were no such plastic effects for flies acclimated at 0°C.

## INTRODUCTION

Ectothermic organisms living in cold environments encounter multiple stressors (cold, dehydration and starvation) which can limit their survival (Block, 1996; Lee, 2010; Rosendale et al., 2017). In cold environments, varying levels of cold conditions are likely to affect stressor-specific plastic responses. For example, larvae of *Drosophila melanogaster* acclimated at 6°C significantly improved their resistance to cold as compared with exposure at 0°C (Kostal et al., 2011). Further, in *D. melanogaster* cold exposure involves plastic changes in some life history traits (survival, longevity and fecundity), which could be a result of depleted energy reserves or accrued tissue damage resulting from chilling (Overgaard et al., 2007; Colinet et al., 2012). Colder environments are also associated with desiccating conditions. Some studies have suggested a physiological link between plastic responses to cold and drought i.e. in freeze-tolerant gall fly *Eurosta solidaginis* (Irwin and Lee, 2002; Williams and Lee, 2008; Levis et al., 2012); in *Belgica antarctica* (Benoit et al., 2009) and in *D. immigrans* (Tamang et al., 2017). In the gall fly *E. solidaginis*, cold-induced changes include increases in the amount and composition of cuticular lipids to decrease water loss; and accumulation of cryoprotectants to reduce the detrimental effects of cold on cellular membranes and proteins (Nelson and Lee, 2004; Lee, 2010; Gantz and Lee, 2015). In contrast, insects face a shortage of food resources during winter which affects energetically expensive processes like reproduction so as to favor the accumulation of body lipids (Angilletta, 2009; Arrese and Soulages, 2010). Therefore, physiological associations between the cold and starvation seem complex.

In insects, levels of body lipids co-vary with starvation resistance (Ballard et al., 2008; Arrese and Soulages, 2010). Interspecific as well as intraspecific analysis of *Drosophila* species support that an increase in starvation resistance is associated with higher storage of body lipids. For example, a comparative study of 23 *Drosophila* species showed threefold interspecific differences in starvation resistance (Matzkin et al., 2009). Another study on five wild-caught populations of *D. simulans* revealed a positive correlation between starvation resistance and body lipids, i.e. twofold higher body lipids in populations from Hawaii and San Diego as compared with populations from Kenya and Australia (Ballard et al., 2008). Ectothermic organisms from temperate regions and/or high altitude localities encounter starvation stress. However, experimental support for an association between resistance to cold and starvation has provided mixed responses. For example, in the freeze-tolerant gall fly *E. solidaginis,* body lipids increased during early autumn by 50% and remained stable during the winter season (Storey and Storey, 1986; Williams and Lee, 2008). In contrast, in the tsetse fly, there is a lack of changes in body lipids in response to cold exposure (Terblanche et al., 2008). In the field, under variable levels of cold conditions, insects are possibly limited in their choice of food resources during autumn and winter. Therefore, acquisition of potential energy metabolites through feeding might affect resistance levels to starvation stress. Thus, in drosophilids, there is a need to examine changes in body lipids due to plastic effects of long-term cold exposure at different thermal conditions which might provide or prevent feeding conditions.

Genetic associations between resistance to cold and starvation have been investigated in laboratory-selected strains for different stressors (cold or heat or desiccation or starvation) in *D. melanogaster* to find cross-tolerance effects (Hoffmann et al., 2005; Bubliy and Loeschcke, 2005). For example, a robust trade-off between resistance to cold and starvation has been evidenced in females but only to a lesser extent in males, based on cold shock mortality in starvation selected lines of *D. melanogaster* (Hoffmann et al., 2005). In contrast, starvation selected lines showed an increase of 16 h in LT_50_ hours for starvation resistance (SR) while cold shock selected lines revealed a lesser increase of ∼5 h in starvation resistance (Bubliy and Loeschcke, 2005). Thus, previous studies on laboratory selected strains of *D. melanogaster* have shown differences in the level of resistance to cold or starvation. Further, cross-tolerance effects of starvation-acclimated flies of *D. melanogaster* did not show changes in chill-coma recovery; and cold-acclimated flies showed no plastic changes in starvation resistance, thereby supporting lack of cross tolerance, but this study did not consider changes in body lipids (Bubliy et al., 2012).

In the present work, we assessed plastic changes in the high altitude *D. immigrans* for adaptations to cold and starvation stress. *D. immigrans* is characterized by large body size, greater tolerance to cold and starvation as well as higher fecundity as compared to *D. melanogaster* (Jenkins and Hoffmann, 1999; Markow and O’ Grady, 2006; Matzkin et al., 2009). *D. immigrans* flies were reared at 15°C and adult flies were exposed to 0°C or 7°C. We assessed cold tolerance (through two metrics i.e. chill-coma recovery; and percent survival due to cold shock at −3°C); and starvation resistance in control and acclimated groups of flies. Previous studies have shown utilization of body lipids under starvation stress while cold-induced plastic changes involve trehalose, glycogen and proline (Rion and Kawecki, 2007; Tamang et al., 2017; Rosendale et al., 2017). Based on this rationale, we analyzed possible accumulation and utilization of four energy metabolites (body lipids, proline, trehalose and glycogen) in *D. immigrans* flies acclimated for different durations (in days) at 0°C or 7°C under fed or non-fed conditions. We compared the rate of utilization of different energy metabolites under cold or starvation stress. We also assessed effects of exposure to cold or starvation on mean daily fecundity of *D. immigrans*. Finally, we examined possible cross protection between cold and starvation based on changes in energy metabolites of *D. immigrans* flies acclimated at 0°C or 7°C.

## RESULTS

### Effects of cold acclimation (0°C or 7°C) on cold resistance

**Fig. 1.**
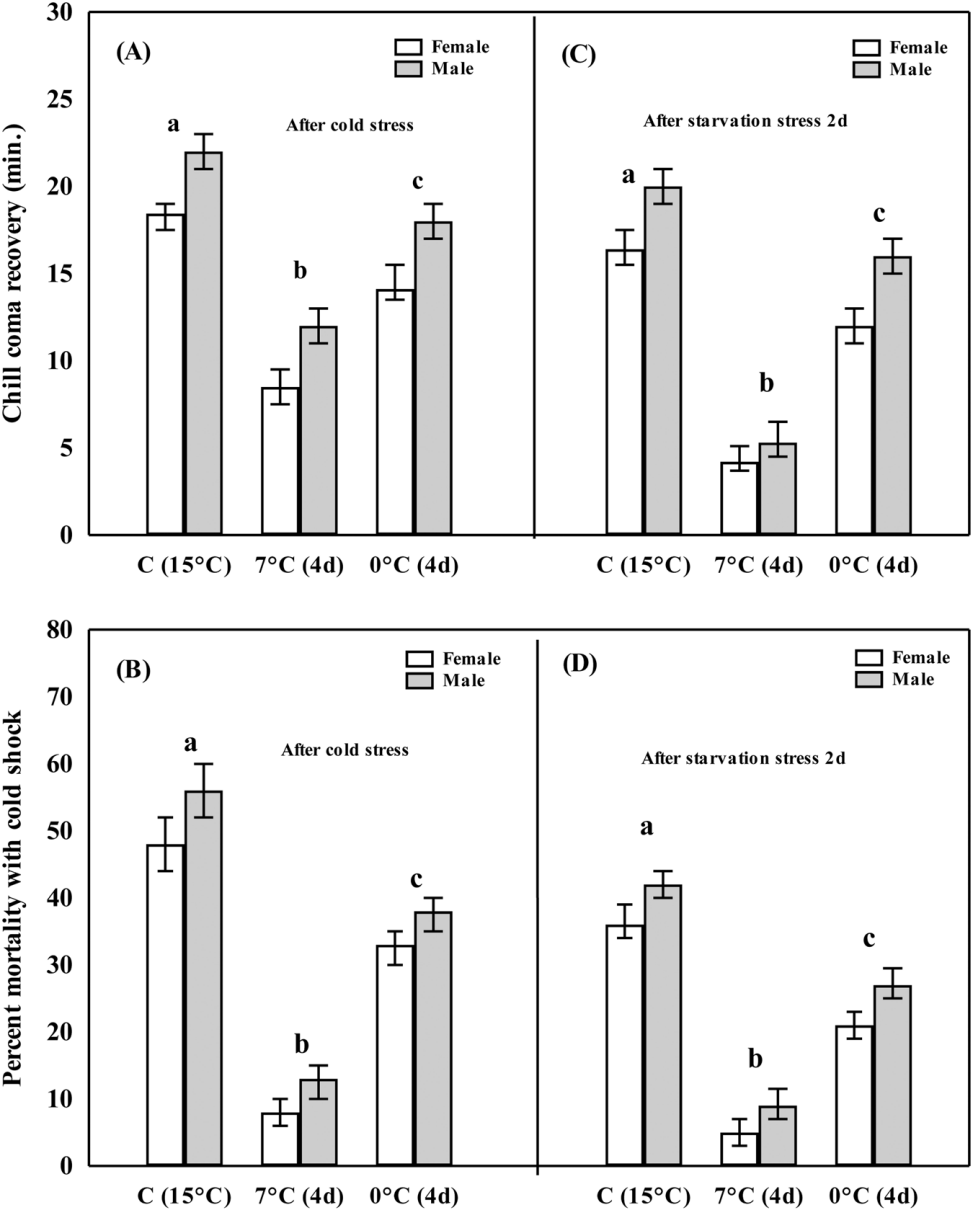
**Plastic changes in cold tolerance due to cold acclimation.** Changes in chill-coma at 0°C or cold-shock mortality at −3°C of control and acclimated flies of both the sexes at 0°C or 7°C for 4 days, and in respective groups of flies after 2 days’ starvation stress. Flies acclimated at 7°C revealed higher cold tolerance than at 0°C. 2 days’ starvation of control and acclimated groups of flies also elicited increased cold tolerance. Different letters indicate significant differences between treatment groups (based on Tukey's test, *P*<0.01).

Cold tolerance of flies acclimated at 0°C or 7°C were significantly higher as compared with control or unacclimated flies (Fig. 1). The recovery duration in the chill-coma assay was inversely proportional to cold tolerance. First, chill-coma recovery duration was significantly lower (8 min) in flies acclimated at 7°C when compared with flies acclimated at 0°C (14 min). However, the recovery time was ∼20 min in the control group of flies (Fig. 1A; Tukey's, *P*<0.01). Second, cold shock mortality at −3°C resulted in 8% mortality of flies acclimated at 7°C as compared to 33% mortality in flies acclimated at 0°C (Fig. 1C; Tukey's, *P*<0.01). Third, we observed a significant increase in cold tolerance in flies from the control group and those cold acclimated at 0°C or 7°C when subjected to 2 days of starvation stress (Fig. 1C,D; Tukey's, *P*<0.01). There was significant reduction in chill-coma recovery when flies acclimated at 7°C were subjected to 2 days of starvation (Fig. 1C; Tukey's, *P*<0.01). Two days' starvation of flies acclimated at 7°C revealed lower cold shock mortality than control as well as flies acclimated at 0°C (Fig. 1D; Tukey's, *P*<0.01). Thus, a cross-tolerance effect was evident because starvation stress was able to improve cold tolerance in flies acclimated at 0°C or 7°C. Finally, results of ANOVA showed significant effects on the basis of acclimation treatments (at 0°C or 7°C) as well as sex (Table S1).

### Plastic changes in body lipids and starvation resistance due to cold acclimation

**Fig. 2.**
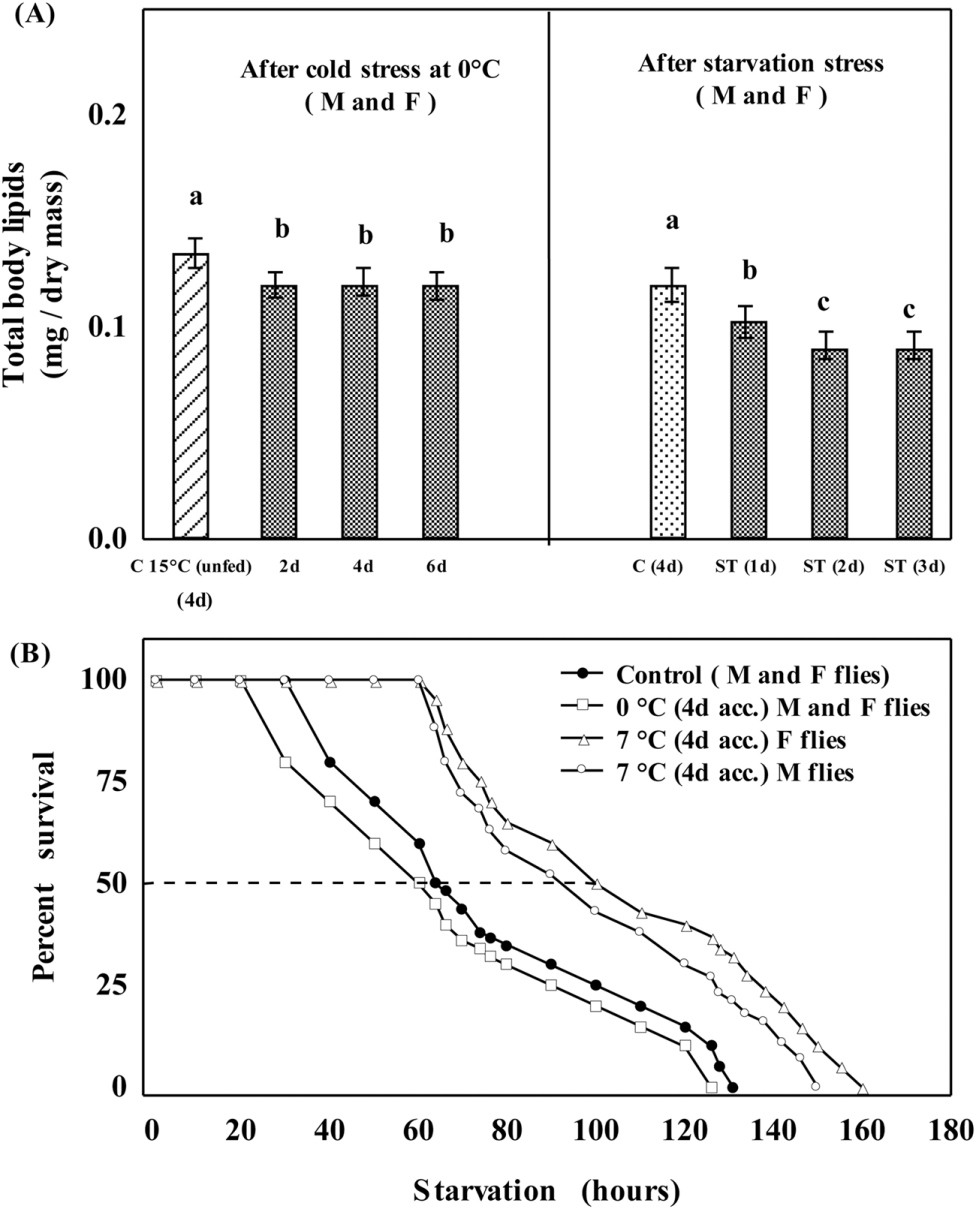
**Plastic changes in body lipids and starvation resistance due to cold acclimation.** Plastic changes in total body lipids of flies acclimated at 0°C (12% reduction up to 2 days in both the sexes; M, male; F, female) and a 28% reduction due to starvation stress in 2 days (A). The control bar in the left panel refers to flies reared at 15°C (unfed for 4 days), while the control bar in the right panel refers to flies acclimated at 0°C for 4 days. Different letters represent significant differences between treatments (Tukey's, *P*<0.01). (B) An increase in starvation survival was evident in flies acclimated at 7°C (with food) but no increase in flies provided with no food. Starvation survival of flies acclimated at 0°C for 4 days was 126 h, compared with 131 h in control group flies.

Flies acclimated at 0°C undergo chill-coma and are unable to feed, while flies acclimated at 7°C are able to feed. Therefore, we compared changes in body lipids in flies acclimated at 0°C or 7°C. Plastic changes in body lipids and starvation resistance of flies cold acclimated at 0°C revealed a 12% reduction in the level of body lipids up to 2 days, while no further reduction in body lipid was evident in flies acclimated at 0°C for 4 or 6 days (Fig. 2A). In contrast, flies acclimated at 7°C for 4 days showed starvation resistance of 160 h as compared with 131 h in control group and 126 h in flies acclimated at 0°C (Fig. 2B).

**Fig. 3.**
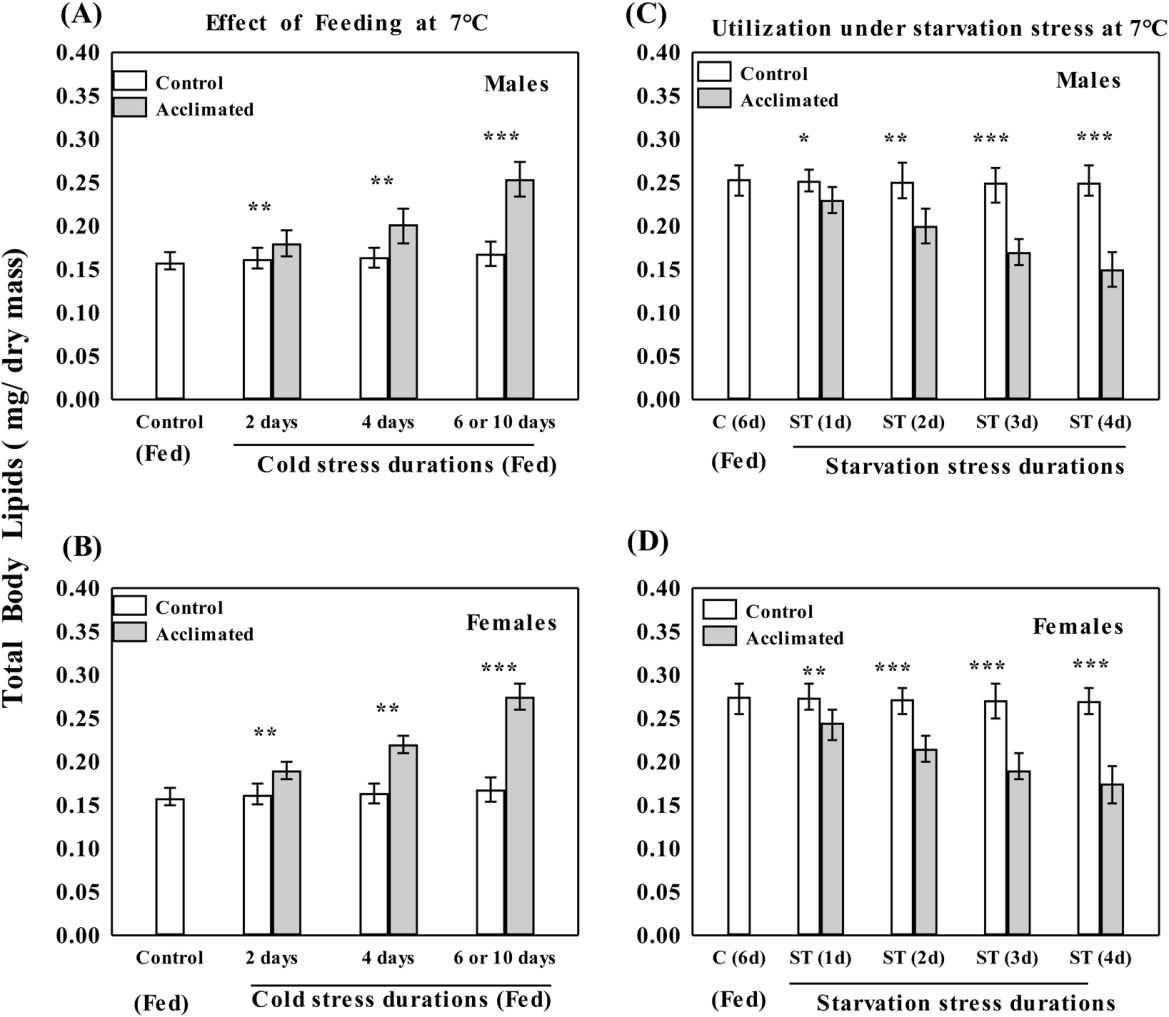
**Plastic changes in body lipids due to feeding at 7°C and utilization under starvation.** Plastic changes for increase of total body lipids in flies acclimated at 7°C (with food) for 2, 4, 6 or 10 days (A,B) while there was no increase in body lipids in flies provided with non-nutritive agar. Age-related changes have been shown for control as well as adults cold acclimated for different durations (2, 4, 6 or 10 days) at 7°C.The utilization of body lipids under different starvation durations of 1, 2, 3 or 4 days at 7°C (C,D) in male and female flies of *D. immigrans.* Statistical difference between each pair of control and acclimated flies was tested with Student's *t*-test (*P<0.05; **P<0.01; ***P<0.001).

Thus, plastic changes for increased level of starvation resistance were evident in flies acclimated at 7°C. Further, we assessed effect of different durations (1–6 days) of feeding at 7°C in order to find corresponding changes in the level of body lipids (Fig. 3). There was a 53% increase in body lipids after 6 days in males (but 63% in females) due to feeding in flies acclimated at 7°C (Fig. 3A,B). Different groups of flies cold acclimated at 7°C for 6 days were subjected to starvation stress (1, 2 or 3 days) and the utilization of lipids are shown in Fig. 3C and D. Thus, increased level of body lipids due to feeding in flies acclimated at 7°C could cope with starvation stress.

### Effects of fed or non-fed conditions on body lipids and ovarian lipids

**Fig. 4.**
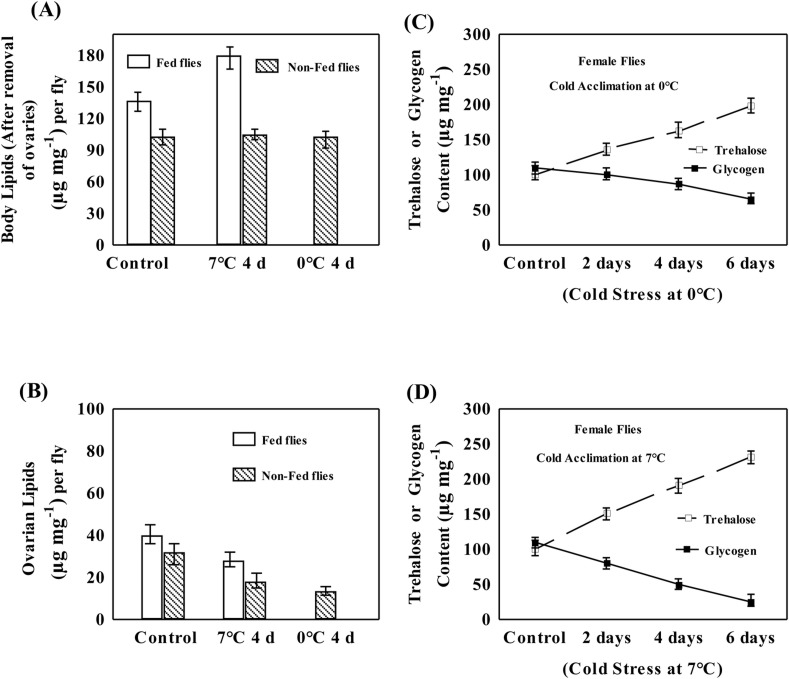
**Changes in the level of body lipids, ovarian lipids; and in trehalose and glycogen in fed or non-fed flies.** (A) Plastic changes in body lipid content minus ovarian lipids; and (B) ovarian lipids of fed and non-fed (non-nutritive agar) female flies of control group reared at 15°C and in flies of *D. immigrans* acclimated at 7°C or 0°C for 4 days. Plastic changes in the levels of trehalose or glycogen induced by 2, 4 or 6 days of cold acclimation at (C) 0°C and (D) 7°C but the patterns did not vary in fed or non-fed flies.

Plastic changes in the levels of total body lipids excluding ovaries; and in the ovaries in flies acclimated at 0°C or 7°C as well as control group are shown in Fig. 4. Body lipids (excluding ovarian lipids) increased significantly (31%) in flies acclimated at 7°C under fed conditions while there was no change in the level of body lipids in non-fed flies acclimated at 0°C or 7°C (Fig. 4A). An interesting observation was that flies acclimated at 7°C (with food) were often seen on food medium as compared to control group of flies reared at 15°C. In contrast, a comparison of ovarian lipids per fly in control versus acclimated groups of flies (0°C or 7°C) revealed effects of feeding conditions (Fig. 4B). The reduction in the levels of ovarian lipids was less in flies acclimated at 7°C as compared to that of 0°C. Thus, adult acclimation at colder conditions (0°C or 7°C) possibly affects feeding of flies and accumulation of body lipids.

### Cold induced changes in trehalose and glycogen

In flies kept on non-nutritive agar, we observed a linear increase in the level of trehalose but decrease in the level of glycogen as a function of different durations of cold acclimation at 0°C or 7°C (Fig. 4C–D). Flies acclimated at 0°C or 7°C showed significant changes in the level of trehalose and glycogen due to cold acclimation (ANOVA, *P*<0.001; Table S1). For flies acclimated at 0°C or 7°C, the fed or non-fed conditions did not affect the patterns of changes in trehalose or glycogen. Thus, plastic changes in trehalose and glycogen are associated with cold acclimation and not with the feeding conditions of the flies.

### Cold or starvation induced changes in the level of proline

**Fig. 5.**
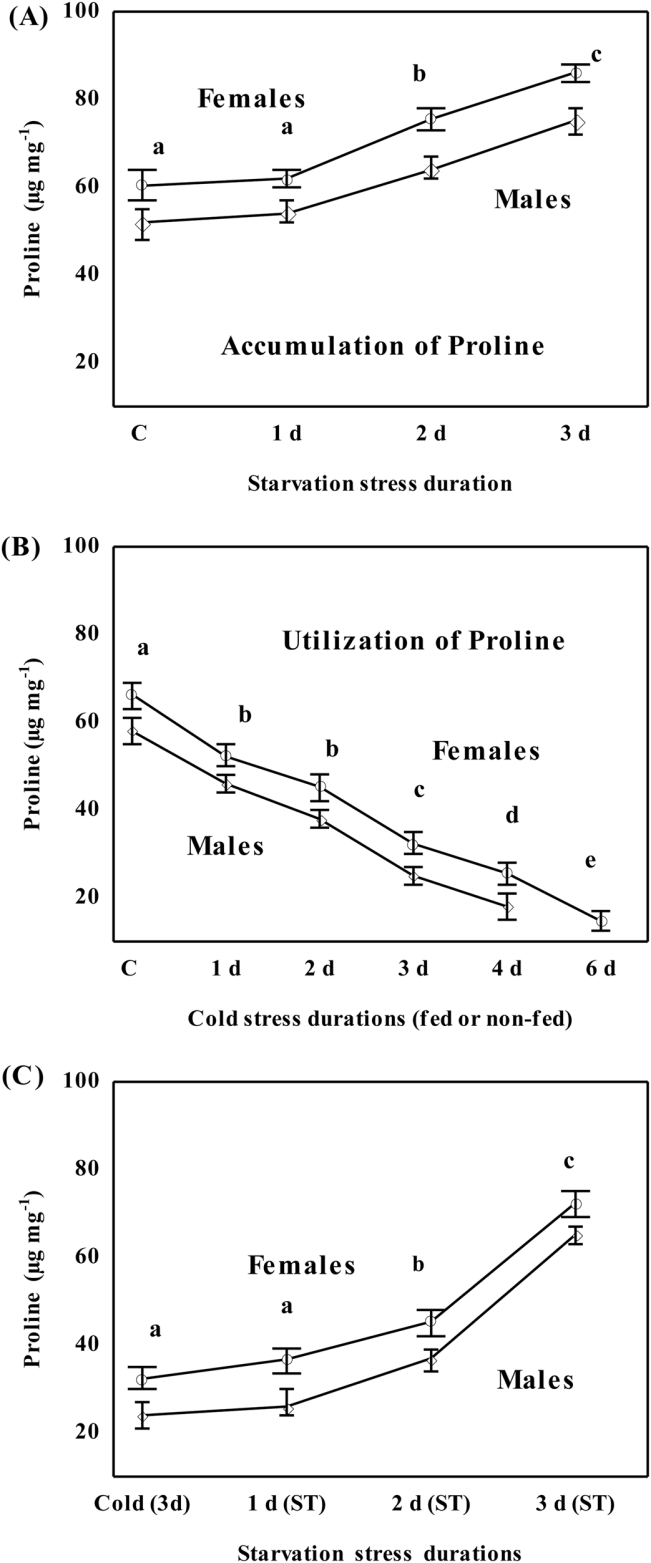
**Plastic changes in the level of proline under starvation or cold stress.** (A) Accumulation of proline under starvation stress durations, and (B) utilization of proline under cold stress durations in *D. immigrans* flies of both sexes (male and female) acclimated at 7°C or 0°C. Changes under starvation stress durations in flies already cold stressed for 3 days are shown in C. Different letters represent significant differences (Tukey's, *P*<0.01) between treatments.

Plastic changes in the level of proline as function of different durations of starvation stress or cold stress are shown in Fig. 5. Starvation increased the level of proline about 1.5-fold after 3 days of starvation as compared to the control group (Tukey's, *P*<0.05; Fig. 5A). The utilization of proline under different durations (1–6 days) of cold stress are shown in Fig. 5B (Tukey's, *P*<0.001). Further, we tested flies with a 50% reduction in proline level after 3 days of cold stress for a possible increase under different durations of starvation. As shown in Fig. 5C, starvation increased the level of proline of cold stressed flies for 3 days (i.e. 30±2.1 to 72.54±2.3 µg mg^−1^; Tukey's, *P*<0.001).

### Plastic changes in energy content in flies acclimated at 0°C or 7°C

Energy content due to body lipids was similar in under fed or non-fed conditions in flies acclimated at 0°C because being in chill-coma, flies were unable to feed. However, in flies acclimated at 7°C (under fed conditions), energy content due to body lipids increased significantly as compared to control group of flies (Table 1). Therefore, an increase in body lipids in flies cold acclimated at 7°C could be due to feeding. Interestingly, a comparison of fed and non-fed flies revealed a 25% increase in energy content due to body lipids in control group of flies (reared at 15°C) when compared with a 65% increase in energy content due to body lipids in case of flies acclimated at 7°C. It may be inferred that cold acclimation at 7°C might increase level of feeding by *D. immigrans*. However, these differences in energy content could be due to 0°C acclimation favoring a different metabolic pathway than 7°C treatment, i.e. biochemical processes could be altered at lower temperatures (0°C or 7°C).

**Table 1.**
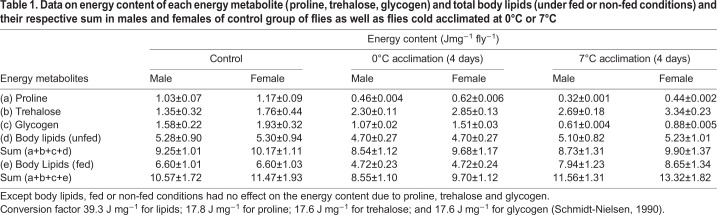
**Data on energy content of each energy metabolite (proline, trehalose, glycogen) and total body lipids (under fed or non-fed conditions) and their respective sum in males and females of control group of flies as well as flies cold acclimated at 0°C or 7°C**

### Stressor specific changes in the rate of change in energy metabolites

Data on regression slope values for changes in the stressor specific accumulation or utilization of total body lipids, proline, trehalose or glycogen in *D. immigrans* groups of flies acclimated at 0°C or 7°C are shown in Table 2. In 0°C acclimated flies, there was no change in total body lipids under different durations of cold or starvation stress. In 7°C acclimated flies, cold stress led to accumulation of body lipids which were utilized under starvation stress (Table 2). An opposite trend was observed for proline, i.e. starvation led to accumulation (+0.36±0.01 µg h^−1^) but cold stress utilized proline (-0.35±0.01 µg h^−1^). In contrast, utilization of proline was evident in both the treatment groups (0°C or 7°C) as a function of different durations of cold stress. Further, a significantly higher rate of accumulation of trehalose (+0.84±0.04 µg h^−1^) was observed for 7°C acclimated flies as compared with +0.65±0.03 µg h^−1^ in 0°C acclimated flies (Table 2). In contrast, there was a significant decrease in the level of glycogen in flies acclimated at 7°C as compared with 0°C (Table 2). It may be noted that starvation stress durations showed no change in the level of trehalose or glycogen (Table 2).

**Table 2.**
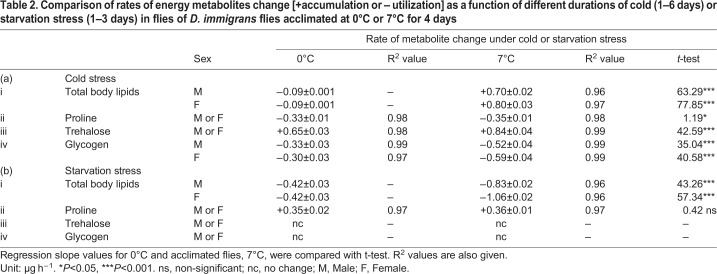
**Comparison of rates of energy metabolites change [+accumulation or – utilization] as a function of different durations of cold (1–6 days) or starvation stress (1–3 days) in flies of *D. immigrans* flies acclimated at 0°C or 7°C for 4 days**

### Effects of cold or starvation acclimation on mean daily fecundity

**Fig. 6.**
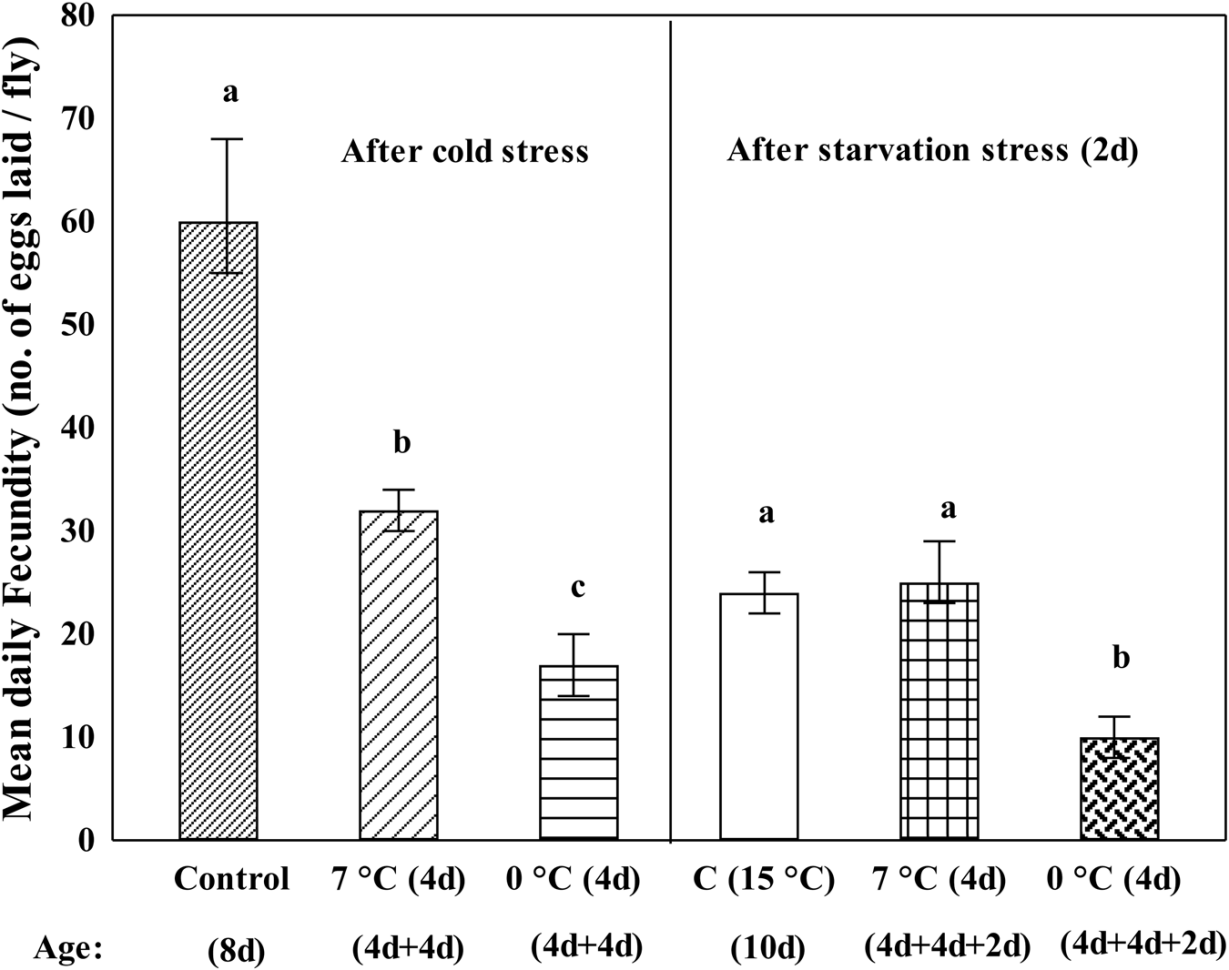
**Comparison of changes in mean daily fecundity per female in control and cold acclimated groups of flies.** Control flies were reared at 15°C and flies acclimated at 0°C or 7°C for 4 days. Mean daily fecundity per fly is also shown for these three groups of flies subjected to starvation stress for 2 days. Reduction in fecundity is lower in 7°C as compared to 0°C acclimated flies of *D. immigrans* (Tukey's, *P*<0.01). For each experiment, the physiological age of flies was kept similar.

Changes in mean daily fecundity of three groups of *D. immigrans* flies (control group of flies reared at 15°C ; flies acclimated either at 0°C or 7°C for 4 days) are shown in Fig. 6. There was a significant reduction in mean daily fecundity in flies acclimated at 0°C (17±3 eggs per day) as compared with the fecundity of control flies (60±5 eggs per day) reared at 15°C (Tukey's, *P*<0.01). However, mean daily fecundity was 32±2 eggs per day in flies acclimated at 7°C. Further, mean daily fecundity was reduced to a third of the level of the control flies after starvation stress for 2 days (Tukey's, *P*<0.01; Fig. 6). Mean daily fecundity after starvation was much reduced (8±1.0 eggs) in 0°C acclimated flies as compared to 25±2 eggs in 7°C acclimated flies (Fig. 6). Thus, mean fecundity of flies acclimated to 7°C was about threefold higher as compared to the 0°C treatment group.

## DISCUSSION

In the high altitude, cold acclimation at 7°C or 0°C increased cold resistance in *D. immigrans*. A comparison of fly groups acclimated for a longer duration (1–6 days) in the presence of food medium or non-nutritive agar revealed feeding at 7°C but no feeding at 0°C. Increases in the level of body lipids were evident in flies acclimated at 7°C under fed conditions only. Therefore, cold acclimation conditions possibly impact the feeding potential of flies to cope with starvation resistance. However, starvation stress (1–3 days) led to an increase in the level of proline which was utilized under cold stress. Cold acclimation at 0°C or 7°C induced increased level of trehalose but decreased the level of glycogen in flies kept on non-nutritive agar. Finally, both cold stress or starvation stress caused greater reduction in mean daily fecundity of flies acclimated at 0°C as compared to flies acclimated at 7°C.

### Plastic changes in cold tolerance

In diverse insect taxa, several studies have shown an increase in cold tolerance due to hardening or acclimation at low or sub-zero temperatures (Storey, 1997; Denlinger and Lee, 2010). In the present work, we selected 7°C because long term acclimation of *D. melanogaster* larvae at 6°C revealed significant (30-fold) cold tolerance as compared to 0°C (Kostal et al., 2011). If cold tolerance is significantly high at 6°C acclimation in *D. melanogaster*, we expect possible plastic changes at 7°C compared with 0°C acclimation in other *Drosophila* species. *D. immigrans* flies acclimated at 7°C significantly increased survival on the basis of chill-coma when compared with plastic changes in flies acclimated at 0°C. Further, cold shock (at –3°C) resulted in significantly lower mortality (8%) in female flies acclimated at 7°C compared with flies acclimated at 0°C (33%). The possible explanation is that long duration acclimation at 0°C would likely lead to indirect chilling injury which might explain the reduced cold tolerance relative to flies acclimated at 7°C. Further, the control group of flies as well as flies acclimated at 0°C or 7°C, subjected to 2 days' starvation, revealed 25–30% reduction in cold shock mortality (Fig. 1). Such observations (based on chill-coma as well as cold-shock mortality) suggest the possible role of starvation in improving cold tolerance of flies acclimated at 0°C or 7°C.

In chill-susceptible *D. melanogaster* and in overwintering insects, several studies have shown significant changes in the level of cryoprotectants (polyols and a range of sugars including trehalose) in response to cold hardening/acclimation (Fields et al., 1998; Irwin and Lee, 2002; Lee, 2010; Colinet et al., 2012). Cold-induced plastic changes in the level of trehalose or other sugars are able to prevent possible damage to cellular membranes and proteins (Denlinger and Lee, 2010). In *D. immigrans*, flies acclimated at 0°C or 7°C resulted in the accumulation of about a twofold higher trehalose content (after 6 days' acclimation) as compared to the control group of flies. We observed cold induced reduction in the level of glycogen in *D. immigrans* flies acclimated at 0°C or 7°C. Cold-induced plastic changes in the levels of trehalose and glycogen were higher in flies acclimated at 7°C than at 0°C. For trehalose or glycogen, plastic changes were similar when acclimation was conducted with food or non-nutritive agar. Therefore, it is likely that trehalose accumulation in *D. immigrans* flies acclimated at 0°C or 7°C involves reshuffling of energy reserves as a consequence of cold acclimation and confers better cold survival. Our observations are consistent with earlier reports on the decrease in glycogen for production of trehalose under stressful cold conditions in *E. solidaginis* (Storey, 1997) and in cabbage armyworm, *Mamestra brassicae L.* (Goto et al., 2001).

### Plastic changes in total body lipids due to cold acclimation

Plastic changes in storage lipids due to developmental or adult acclimation, and in different thermal conditions have received less attention in *Drosophila* species (Rion and Kawecki, 2007). Developmental acclimation affected the level of body lipids in some insects, e.g. cold adapted aphid parasitoid *Aphidius colemani* stores more body lipids when grown at cooler rather than warmer temperatures (Colinet et al., 2007). In overwintering freeze-tolerant gall fly larvae of *E. solidaginis*, the levels of body lipids increased during the autumn (Storey and Storey, 1986). These studies have shown plastic changes in the level of body lipids due to different rearing thermal conditions. In *D. immigrans* (flies acclimated at 0°C or 7°C) significant differences in the levels of body lipids depend upon fed or non-fed conditions of the flies. In flies acclimated at 7°C (under fed condition only), cold acclimation might influence the feeding potential to build the levels of body lipids. Thus, cold acclimation conditions (0°C or 7°C) possibly constrain feeding potential, thereby affecting the levels of body lipids in *D. immigrans.*

### Proline provides a link between resistance to cold and starvation

Proline is an energy dense metabolite because energy yield from its partial oxidation is only slightly lower in comparison to lipids (Renault et al., 2006). Accumulation of proline as a consequence of cold acclimation has been shown in the beetle *Sitophilus granaries* (Fields et al., 1998); *D. melanogaster* (Misener et al., 2001; Kostal et al., 2011) and in the overwintering gall fly *E. solidaginis* (Storey et al., 1981). Interspecific differences in adaptations to cold are associated with proline levels, i.e. the European corn borer *Ostrinia nubilalis* accumulates more proline than the south-western corn borer *Diatraea grandiosella* (Morgan and Chippindale, 1983). A comparative study on proline levels showed lack of proline in fresh water species of Hemiptera, while terrestrial species exhibited higher levels of proline (Singh, 1978). However, associations between proline and starvation have been less investigated in different insects. For example, in the Colorado potato beetle *Leptinotarsa decemlineata*, starvation for 1–3 days led to a 40% increase in proline levels in body fat as well as flight muscles (Weeda and De Kort, 1979); and in the flight muscles of locusts (Goldsworthy and Cheeseman, 1978). In contrast, there is paucity of information on changes in proline levels under starvation stress in drosophilids. This could be due to a greater focus on the utilization of storage lipids under starvation stress, while changes in free amino acids received lesser attention (Rion and Kawecki, 2007). In the present study we observed plastic responses (accumulation of proline under starvation stress but utilization under cold stress) in *D. immigrans* flies reared at 15°C and acclimated at 0°C or 7°C. This is an interesting observation which supports a link between resistance to cold and starvation and needs to be explored in diverse insect taxa.

### Cost of cold acclimation (0°C or 7°C) or starvation on fecundity

In insects, fitness related traits such as fecundity, egg viability and other life history traits are constrained due to multiple stressors (Angilletta, 2009). Among drosophilids, *D. melanogaster* has received greater attention as compared to cold- or warm-adapted *Drosophila* species. A trade-off between resistance to cold and mean daily fecundity has been evidenced in *D. melanogaster*, i.e. a 60% reduction in offspring production was observed in flies subjected to rapid cold hardening while cold shock at –5°C, resulted in an almost total loss of offspring production (Overgaard et al., 2007). For drosophilids, starvation is likely to impact mean daily fecundity but has received less attention. *D. immigrans* flies acclimated at 0°C revealed significant reduction in mean daily fecundity as compared with flies acclimated at 7°C. Such plastic changes in mean daily fecundity are consistent with a greater reduction of ovarian lipids in flies acclimated at 0°C as compared to 7°C (Fig. 4). In contrast, starvation (2 day) caused a significant reduction in the mean daily fecundity of flies acclimated at 0°C compared to control and 7°C acclimated flies (Fig. 6). In terms of mean daily fecundity, flies acclimated at 0°C had a significantly higher cost compared to flies acclimated at 7°C after cold or starvation stress. Thus, *D. immigrans* flies acclimated at 7°C showed significantly higher survival under cold or starvation stress, as well as higher mean daily fecundity.

### Conclusion

In colder environments, *Drosophila* species encounter cold and starvation stress simultaneously and adaptations to these stressors might involve plastic responses. In the present work major observations include (a) cold acclimation at 7°C revealed higher survival under cold shock at –3°C than 0°C acclimated flies; (b) an increase in the levels of body lipids was evident in flies cold acclimated at 7°C (under fed conditions only) but not in flies acclimated at 0°C, which remained unfed due to chill-coma; (c) starvation stress durations elicited proline that was utilized under cold stress, which might favor cross-protection between cold and starvation; (d) cold acclimation at 0°C or 7°C (in non-fed flies) led to an accumulation of trehalose but a reduction in the level of glycogen. However, possible reshuffling between glycogen and trehalose was higher in flies cold acclimated at 7°C as compared to 0°C; (e) mean daily fecundity under cold or starvation was significantly higher in 7°C acclimated flies compared with 0°C. Therefore, plastic changes in the level of trehalose and glycogen are associated with cold acclimation while an increase in the levels of body lipids depends upon possible increased feeding at 7°C. Thus, plastic changes in body lipids vary depending upon the cold acclimation conditions.

## MATERIALS AND METHODS

### Collection and cultures

Wild-caught individuals (*n* ∼200 flies) of *D. immigrans* were collected during mid-October 2016 (pre-winter season) from a highland locality of western Himalayas (Shimla: altitude 2206 m; latitude 31.61°N; longitude 77.10 °E; T_avg_ 13–17°C; RH 46–52%). Cultures were maintained at low density (about 30 pairs) on standard cornmeal-yeast-agar medium following Markow and O’ Grady (2006) in 300 ml culture bottles. The flies were reared under season specific thermal and humidity conditions (15±0.5°C; RH 50±2%; 12L:12D); and both male and female adult flies of *D. immigrans* were used for assessment of different traits. We investigated plastic changes in tolerance to cold and starvation as well as energy metabolites in both the sexes of *D. immigrans* reared at 15°C followed by acclimation of adult flies at 0°C or 7°C.

### Experimental set-up

*D. immigrans* flies reared at 15°C were used as the control group. For cold acclimation at 0°C or 7°C for 1–6 days, flies were kept in vials having either *Drosophila* standard food medium (fed) or non-nutritive agar (non-fed). We also considered age-related changes in the level of body lipids in the control group as well as the acclimated groups of flies. Flies of different acclimation treatments were subjected to starvation stress for 1–3 days at the respective acclimation temperatures. We simultaneously analyzed control flies and flies acclimated at 0°C, 7°C (under fed or non-fed conditions) for changes in resistance to cold and starvation. These three groups of flies, under fed or non-fed conditions, were also assayed for four energy metabolites (body lipids, proline, trehalose and glycogen). Further, we assessed plastic changes in mean daily fecundity in flies in the control group as well as flies acclimated at 0°C or 7°C followed by 12 h recovery at 15°C, and also after 2 days' starvation at the respective acclimation temperatures.

### Assessment of resistance to cold and starvation

#### Cold tolerance

We measured resistance to cold through two different methods, i.e. chill-coma recovery assay at 0°C and cold shock mortality at –3°C. Adult flies were acclimated for 4 days at 0°C or 7°C in Tarson's plastic vials (37×100 mm; http://www.tarsons.in) containing *Drosophila* food medium or non-nutritive agar in the cap (35×15 mm) while flies were kept at the base of the vial. For chill-coma assay, these adult flies were subjected to 0°C for 48 h followed by an estimation of their recovery (duration in minutes) until they were able to right themselves (Bubliy et al., 2012). Further, for cold shock assay, adult flies were subjected to –3°C for 16 h and we recorded the number of surviving individuals after 20 h, i.e. when the flies were able to walk (Overgaard et al., 2007). Flies cold acclimated at 0°C (4 days) or 7°C (4 days) were also subjected to starvation stress (2 days) at 0°C or 7°C followed by estimation of cold tolerance. For each experiment, three replicates of 30 flies were tested for cold tolerance assays.

#### Starvation resistance

Starvation resistance was measured while flies were kept at 0°C or 7°C for different durations of starvation stress. Starvation resistance of each group (control or acclimated) was analyzed in three replicates of 30 flies. For each replicate – three tubes with ten flies each – starvation resistance was measured as survival time till death under wet conditions (85–90% RH) but without food. Starvation assay set up involved two plastic vials, each measuring 10 cm×3 cm. The lower vial contained foam sponge impregnated with 4 ml of water (+2 mg sodium benzoate to prevent any bacterial growth) while the upper inverted tube (having non-nutritive agar medium at the bottom) had ten adult flies and was kept on top of the lower tube covered with muslin cloth and by cello-taping around the mouth of both tubes. The mortality time was recorded twice a day (08:00 and 20:00) until all flies had died from starvation. For estimation of starvation survival duration, the two vials experimental setup used in this study is likely to minimize the loss of body water.

### Mean daily fecundity

We analyzed the possible cost of cold or starvation treatments on mean daily fecundity in control as well as groups of flies acclimated at 0°C or 7°C followed by 12 h recovery at 15°C. For estimating fecundity, virgins were collected from each culture vial early in the morning (08:00). One virgin female and one virgin male were kept in independent mating chambers for 24 h. The flies were transferred to fresh food vials every day, and the number of eggs laid by each female after 24 h was recorded daily for 10 days. For these experiments, live yeast was not provided in the food medium. Fecundity of *D. immigrans* (reared at 15°C) was estimated in different groups of flies which were cold acclimated at 0°C (4 days), 7°C (4 days) and those subjected to starvation stress (2 days). We also analyzed the effect of starvation (2 days) on mean daily fecundity of flies cold acclimated at 0°C or 7°C. For each set of experiments, three replicates of ten pairs of flies were used for mean daily fecundity following Overgaard et al. (2007).

### Estimation of body and ovarian lipids

For lipid content, each individual fly was dried in a 2 ml Eppendorf tube (http://www.tarsons.in) at 60°C for 48 h and then weighed on Sartorius microbalance (Model-CPA26P; 0.001 mg precision; http://www.sartorius.com/). Thereafter, 1.5 ml diethyl ether was added in each Eppendorf tube and kept for 24 h under continuous shaking (200 rpm) at 37°C. After decanting the medium, fresh 1.5 ml diethyl ether was added again and kept for 24 h under continuous shaking (200 rpm) at 37°C. Finally, the solvent was removed and individuals were again dried at 60°C for 48 h and reweighed. Similarly, for assessment of body lipids and ovarian lipids in individual female flies of control as well as acclimated groups (under fed or non-fed conditions), we isolated a pair of ovaries by pulling the tip of the female abdomen from the rest of body with the help of fine-tipped forceps. For each female fly, separated ovaries and the rest of the body were kept in different Eppendorf tubes followed by drying at 60°C for 2 days to obtain dry mass. We estimated body lipids separately in the ovary and also in the rest of body. Ovarian lipids, as well as body lipids, were independently estimated as initial dry mass – lipid free dry mass following Hoffmann et al. (2005). For calculating rate of change in body lipids, flies were subjected to cold acclimation at 0°C (1 day, 2 days, 4 days, and 6 days) or 7°C (2 days, 4 days and 6 days), and starvation stress (1 day, 2 days and 3 days) of *D. immigrans* flies acclimated at 0°C (4 days) and 7°C (6 days).

### Proline estimation

Proline content in fly homogenates was determined by the modified method following Bergman and Loxley (1970). In this assay, interference from primary amino acids was eliminated by nitrous acid treatment and the excess nitrous acid was removed by heating with ammonium chloride followed by hydrochloric acid. Interfering materials are also removed by their absorption into the protein-sulphosalicylic acid complex. Proline content was estimated in each of the three replicates of 30 flies of each group and sex.

The 30 adult flies were homogenized in 3 ml of sulphosalicylic acid. Following centrifugation, 50 μl of the homogenate was added to 15 μl of freshly prepared 1.25 M sodium nitrite solution and the contents were mixed and kept at room temperature for 20 min. Further, 15 μl of 1.25 M ammonium chloride solution was added and the contents were mixed followed by an addition of 60 μl of concentrated hydrochloric acid. The contents were mixed and heated in a boiling water bath for 20 min. The tubes were cooled and 60 μl of 10 N sodium hydroxide was added. To the resulting solution, we added 200 μl glacial acetic acid and 200 μl of ninhydrin solution in each capped tube. The solutions were then mixed and incubated for 60 min. at 100°C. Following incubation, the samples were extracted with toluene, and absorbance of the aqueous phase was quantified spectro-photometrically at 520 nm and the amount of proline was estimated in reference to a standard curve. Further, the rate of accumulation of proline was measured as a consequence of different durations of starvation stress (1 day, 2 days and 3 days) for flies acclimated at 0°C or 7°C. However, the rate of utilization of proline was estimated in flies exposed to different durations (1 day, 2 days, 3 days, 4 days and 6 days) of cold stress (0°C or 7°C).

### Trehalose and Glycogen estimation

Estimation of trehalose and glycogen content were made in *D. immigrans* flies from the control group as well as the treatment groups. For sample preparation, each of the three replicates of 30 flies of each group and sex were homogenized in a homogenizer (Labsonic^®^ M; http://www.sartorius.com/) with 300 μl Na_2_CO_3_ and incubated at 95°C for 2 h to denature proteins. An aqueous solution of 150 μl acetic acid (1 M) and 600 μl sodium acetate (0.2 M) was mixed with the homogenate. Thereafter, the homogenate was centrifuged (Fresco 21, Thermo-Fisher Scientific) at 12,000 rpm (9660×***g***) for 10 min. This homogenate was used for independent estimations of trehalose and glycogen. For trehalose assay, aliquots (200 μl) were placed in two different tubes; one was taken as a blank whereas the other was digested with trehalase at 37°C using the Megazyme trehalose assay kit (K-Treh 10/10, http://www.megazyme.com). In this assay, released D-glucose was phosphorylated by hexokinase and ATP to glucose-6-phosphate and ADP, which was further coupled with glucose-6-phosphate dehydrogenase that resulted in the reduction of nicotinamide adenine dinucleotide (NAD). The absorbance by NADH was measured at 630 nm (UV-2450-VIS, Shimadzu Scientific Instruments, Columbia, USA). The pre-existing glucose level in the sample was determined in a control reaction lacking trehalase and subtracted from total glucose concentration (Tamang et al., 2017).

For estimation of glycogen content, a quantity of 50 µl aliquot was incubated with 500 µl *Aspergillus niger* glucoamylase solution (8.7 U ml^−1^ in 200 mM of acetate buffer) for 2 h at 40°C with constant agitation and the suspension was centrifuged at 4000 rpm for 5 min. It mainly hydrolyzed alpha (1–4) and alpha (1–6) glycosyl linkages and was suited for breakdown of glycogen. Glucose concentration was determined with 20 µl of supernatant from the suspension and added with 170 µl of a mixture of G6-DPH (0.9 U ml-1); ATP (1.6 mM); and NADP (1.25 mM) in triethanolamine hydrochloride buffer (380 mM TEA–HCl and 5.5 mM of MgSO4) and 10 µl of hexokinase solution (32.5 U ml-1 in 3.2 M ammonium sulfate buffer), and absorbance was measured at 625 nm (Marron et al., 2003). Further, the rate of accumulation or utilization of trehalose or glycogen was measured as a function of different durations of cold or starvation stress in flies kept with *Drosophila* standard food medium (fed) or non-nutritive agar (non-fed). The rate was estimated for each group in three replicates of 30 flies.

### Statistical analysis

Data on mean±s.e. of chill-coma recovery, as well as of percentage mortality due to cold shock of three groups of 30 flies of control and treatment groups, were shown as bar diagrams. Data on basal level (control) and acclimated flies (0°C or 7°C) were subjected to two-way ANOVA for analysis of effects due to treatment, sex and their interactions in Table S1. The energy content (body lipids, proline, trehalose and glycogen) was calculated using standard conversion factors (Schmidt-Nielsen, 1990). The amount of each energy metabolite was multiplied by conversion factor, i.e. for body lipid (39.3 Jmg^−1^); proline (17.8 Jmg^−1^); trehalose and glycogen (17.6 Jmg^−1^). For the analysis of rate of accumulation or utilization of each energy metabolite we calculated regression slope values and differences in slope values were compared with Student's *t*-test (Table 2). Data on mean fecundity per day of flies acclimated at 0°C or 7°C were compared with the control group. For statistical comparison we used different superscript letters on the basis of Tukey's test. Statistical calculations and illustrations were made with the help of Statistica 7.

## Supplementary Material

Supplementary information
