# Supplementary material for: Cold acclimation conditions constrain plastic responses for resistance to cold and starvation in Drosophila immigrans
Source: Biol Open. 2018 Jun 15;7(6):bio034447. doi: 10.1242/bio.034447 (PMC6031344; doi:10.1242/bio.034447)
Supplement: Supplementary information [file biolopen-7-034447-s1.pdf]

Table S1. Results of two-way ANOVA for assessing the effects of treatment (control vs acclimated), sex and their interaction for three stress related traits (CCR, Cold Shock Mortality and ST) and four energy metabolites (Lipids, Proline, Trehalose and Glycogen) in *D.immigrans* reared at 15°C (control) and groups of flies acclimated at 0°C or 7°C for 4 days.

			Treatment (T)	Sex (S)	T × S	Error
	Traits	df	2	1	2	114
1.	Chill coma recovery	MS	835.11	528.82	6.23	2.49
	(CCR, minutes)	F	334.79***	212***	2.50*	
		% Var.	66.92	21.18	0.49	11.39
2.	Cold shock mortality	MS	17235.2	1346.7	59.0	2.7
		F	6501.69***	508.02***	22.27***	
		% Var	95.12	3.71	0.32	0.83
3.	Starvation resistance	MS	9778	464	376	3
	(ST, h)	F	3118.0***	148.0***	119.9***	
		% Var.	92.55	2.19	3.55	1.69
4.	Total body lipids	MS	84200	822	1157	6
	(µg mg^{-1}) fed flies	F	12957.3***	126.4**	178**	
		% Var.	97.69	0.95	1.34	0.007
5.	Total body lipids	MS	2779	14	36	5
	(µg mg^{-1}) non-fed	F	609.0***	3.1ns	7.8***	
	flies	% Var.	90.17	0.22	1.15	8.44
6.	Proline	MS	18011.2	2225.6	19.1	3.4
	(µg mg^{-1})	F	5355.61***	661.78***	5.67***	
		% Var.	93.15	5.75	0.09	0.99
7.	Trehalose	MS	70406	22647	452	7
	(µg mg^{-1})	F	10430.2***	3355.0***	66.90***	
		% Var.	85.68	13.71	0.54	0.46
8.	Glycogen	MS	33185.2	12353.8	290.5	8.5
	(µg mg^{-1})	F	8910.37***	1455.70***	34.23***	
		% Var.	82.68	15.38	0.72	1.20

*p< 0.05;**p<0.01; ***p<0.001; ns = non-significant
